# Inhibition of Wnt/β‐catenin signaling upregulates Na_v_1.5 channels in Brugada syndrome iPSC‐derived cardiomyocytes

**DOI:** 10.14814/phy2.15696

**Published:** 2023-05-24

**Authors:** Aizhu Lu, Ruonan Gu, Cencen Chu, Ying Xia, Jerry Wang, Darryl R. Davis, Wenbin Liang

**Affiliations:** ^1^ University of Ottawa Heart Institute Ottawa Ontario Canada; ^2^ Department of Cellular and Molecular Medicine University of Ottawa Ottawa Ontario Canada; ^3^ Department of Anesthesiology, Zhujiang Hospital Southern Medical University Guangzhou China

**Keywords:** cardiomyocytes, induced pluripotent stem cells, Na_v_1.5, Wnt/β‐catenin signaling

## Abstract

The voltage‐gated Na_v_1.5 channels mediate the fast Na^+^ current (*I*_Na_) in cardiomyocytes initiating action potentials and cardiac contraction. Downregulation of *I*_Na_, as occurs in Brugada syndrome (BrS), causes ventricular arrhythmias. The present study investigated whether the Wnt/β‐catenin signaling regulates Na_v_1.5 in human‐induced pluripotent stem cell‐derived cardiomyocytes (iPSC‐CMs). In healthy male and female iPSC‐CMs, activation of Wnt/β‐catenin signaling by CHIR‐99021 reduced (*p* < 0.01) both Na_v_1.5 protein and *SCN5A* mRNA. In iPSC‐CMs from a BrS patient, both Na_v_1.5 protein and peak *I*_Na_ were reduced compared to those in healthy iPSC‐CMs. Treatment of BrS iPSC‐CMs with Wnt‐C59, a small‐molecule Wnt inhibitor, led to a 2.1‐fold increase in Na_v_1.5 protein (*p* = 0.0005) but surprisingly did not affect *SCN5A* mRNA (*p* = 0.146). Similarly, inhibition of Wnt signaling using shRNA‐mediated β‐catenin knockdown in BrS iPSC‐CMs led to a 4.0‐fold increase in Na_v_1.5, which was associated with a 4.9‐fold increase in peak *I*_Na_ but only a 2.1‐fold increase in *SCN5A* mRNA. The upregulation of Na_v_1.5 by β‐catenin knockdown was verified in iPSC‐CMs from a second BrS patient. This study demonstrated that Wnt/β‐catenin signaling inhibits Na_v_1.5 expression in both male and female human iPSC‐CMs, and inhibition of Wnt/β‐catenin signaling upregulates Na_v_1.5 in BrS iPSC‐CMs through both transcriptional and posttranscriptional mechanisms.

## 1 INTRODUCTION

Voltage‐gated Na^+^ current (*I*_Na_) in cardiomyocytes is responsible for the rapid upstroke of action potentials and their fast conduction, which are crucial for the coordinated contraction of the heart (Marban, 2002). Reduction in *I*_Na_ is associated with ventricular remodeling in heart disease and contribute to pathogenic cardiac rhythms. Reduced cardiac *I*_Na_ is found in pigs after myocardial infarction (Pu & Boyden, 1997), in heart failure patients (Shang et al., 2007; Valdivia et al., 2005), and in dogs with heart failure (Maltsev et al., 2002). Reduced *I*_Na_ causes slow conduction (Park et al., 2015) and induces lethal ventricular arrhythmias (Papadatos et al., 2002; Park et al., 2015).

Reduced *I*_Na_ is also responsible for the most common type of Brugada syndrome (BrS, Type 1) (Brugada & Brugada, 1992; Kapplinger et al., 2010) and a portion of the early repolarization (ER) syndrome (Antzelevitch & Yan, 2015; Watanabe et al., 2011), both of which are associated with malignant ventricular arrhythmias and sudden deaths but no disease‐specific therapies are available. BrS and ER syndromes are two forms of the J‐wave syndrome (Antzelevitch & Yan, 2015) and differ in the magnitude and location of J‐waves on surface ECG. Reduced *I*_Na_ exaggerates the transmural dispersion of repolarization in ventricular myocardium, manifested in ECG as J‐waves (deflections immediately after the QRS complexes) (Antzelevitch & Yan, 2010) and triggers ventricular tachyarrhythmias. Type 1 BrS is caused by mutations in *SCN5A* gene (encoding the pore‐forming α subunit of cardiac *I*_Na_, Na_v_1.5) leading to *I*_Na_ reductions via various mechanisms (Kapplinger et al., 2010; Meregalli et al., 2009; Tan et al., 2003; Vatta et al., 2002; Wang et al., 2000). Recent studies have also found a lower expression level of *SCN5A*/Na_v_1.5 in BrS cardiomyocytes (Bersell et al., 2022; Gaborit et al., 2009; Liang et al., 2016), suggesting that reduced Na_v_1.5 channel expression may be an additional pathogenic mechanism. Previous studies have shown that slowing Na_v_1.5 channel inactivation with dimethyl lithospermate B (extracted from Chinese herbs) increased *I*_Na_ and attenuated arrhythmias in a pharmacologically induced Brugada model (Fish et al., 2006). However, strategies to increase Na_v_1.5 level for correcting the reduced *I*_Na_ in BrS are lacking.

**FIGURE 1:**
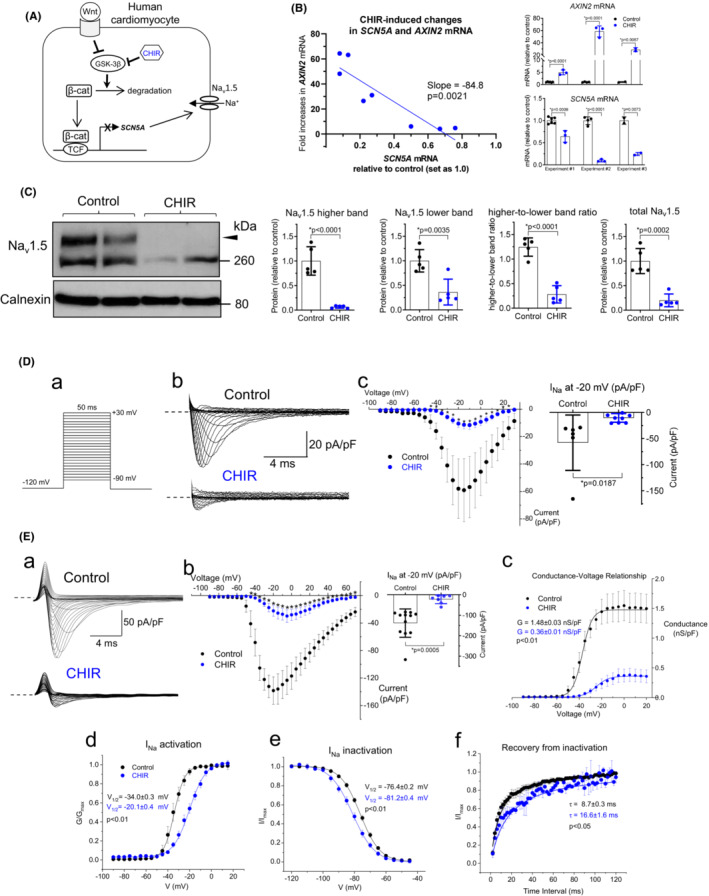
Wnt/β‐catenin signaling reduces *SCN5A* mRNA, Na_v_1.5 protein, and *I*_Na_ in male iPSC‐derived cardiomyocytes. (A) Diagram of the Wnt/β‐catenin pathway which can be activated by CHIR that inhibits GSK‐3β, a key mediator of β‐catenin (β‐cat) degradation; β‐catenin translocates into nucleus and, together with TCF, regulates the transcription of target genes. GSK‐3β, glycogen synthase kinase 3β; TCF, T‐cell factor. (B) qRT‐PCR showing correlation between reductions in *SCN5A* mRNA and increases in *AXIN2* mRNA in male healthy iPSC‐CMs after treatment with CHIR for 48 h (5 μM, *n* = 8 samples from three differentiations). Data were normalized to values in vehicle (DMSO)‐treated cells (*n* = 12 samples from three differentiations). The smaller changes in *AXIN2* and *SCN5A* mRNA in experiment #1 are likely due to the reduced activity of the CHIR molecules after prolonged storage (>6 months) in stock solution. Data were analyzed by two‐tailed *t*‐test. (C) Left panel: representative western blot showing reduced Na_v_1.5 (using a polyclonal antibody) in male iPSC‐CMs after treatment with CHIR for 48 h. Calnexin was used as a loading control. Right panels show quantification of Na_v_1.5 higher band, lower band, the higher‐to‐lower band ratio, and total Na_v_1.5 (both bands). For both groups: *n* = 5 samples from two differentiations. Data were analyzed by two‐tailed *t*‐test. (D) *I*_Na_ recorded from male iPSC‐CMs with a bath solution containing 20 mM Na^+^ (*E*_Na_ = +35 mV) and 0.1 mM Cd^2+^ (to inhibit L‐type Ca^2+^ current). (a) Voltage protocol used to elicit *I*_Na_. Cells were held at −120 mV and *I*_Na_ was elicited by 50‐ms voltage steps ranging from −90 to +30 mV with 5‐mV increments. (b) Representative *I*_Na_ recorded from healthy iPSC‐CMs cultured in control medium (containing DMSO, top panel) or in medium containing 5 μM CHIR (bottom panel) for 48 h. Dashed lines indicate zero current. (c) Left, current–voltage relationship of *I*_Na_ in DMSO‐treated (control, *n* = 6 cells from two batches of cells, black dots) and CHIR‐treated (*n* = 9 cells from two differentiations of cells, blue dots) iPSC‐CMs. *p < 0.05 versus control by two‐tailed *t*‐test. Error bars indicate standard errors of the mean for clarity. Right panel shows individual data points at −20 mV. Error bars indicate standard deviations. (E) *I*_Na_ recorded from male iPSC‐CMs with a bath solution containing 100 mM Na^+^ (*E*_Na_ = +76 mV) without Cd^2+^ (instead, nifedipine was included to inhibit L‐type Ca^2+^ current). Error bars indicate standard errors of the mean for clarity. (a) Representative *I*_Na_ recorded from iPSC‐CMs cultured in control medium containing DMSO (top panel) or in medium containing 5 μM CHIR for 48 h (bottom panel). (b) Current–voltage relationship of *I*_Na_ in DMSO‐treated (control, *n* = 12 cells from two differentiations, black dots) and CHIR‐treated (*n* = 7 cells from two differentiations of cells, blue dots) iPSC‐CMs. *p < 0.01 versus control by two‐tailed *t*‐test. Right panel shows individual data points at −20 mVand error bars indicate standard deviations. (c) *I*_Na_ conductance‐voltage relationship derived from data shown in panel Eb. Conductance was calculated by dividing the current density by the driving force (*E*_m_ – Reversal Potential) for each testing voltage (*E*_m_). The two curves (black for control, *n* = 8 cells and blue for CHIR, *n* = 7 cells) were fitted with Boltzmann equation: y = A2 + (A1–A2)/(1 + exp((x–x0)/dx)) where A2 is the conductance in the unit of nano Siemens per pico Farad (nS/pF). (d) Steady‐state activation curves. Colored curves (black for control and blue for CHIR) were fitted with Boltzmann equation: as shown above where x0 is the *E*_m_ for half‐maximal activation of *I*_Na_ (voltage for 50% activation, *V*_1/2_), and dx is the *k* value (slope factor). *V*_1/2_ were −34.0 ± 0.3 mV in control (*n* = 8 cells) and − 20.1 ± 0.4 mV in CHIR group (*n* = 7 cells). *k* values were 4.8 ± 0.1 in control and 7.5 ± 0.3 in CHIR group (*p* < 0.01). (e) *I*_Na_ steady‐state inactivation curves. Cells were held for 1 second at different potentials from −120 to −45 mV before a step to 0 mV to elicit *I*_Na_. Voltages for 50% inactivation (*V*_1/2_) were − 76.4 ± 0.2 mV for control (*n* = 14 cells) and − 81.2 ± 0.4 mV for CHIR (*n* = 5 cells, *p* < 0.01). *k* values were 6.3 ± 0.1 in control and 7.1 ± 0.3 in CHIR group (*p* < 0.05). (f) Recovery from inactivation. Two 20‐ms pulses to −20 mV were applied with an interpulse potential of −120 mV at different intervals with 2‐ms increments. Time for 50% recovery, *t* = 16.6 ± 1.6 ms for CHIR *n* = 5 cells versus 8.7 ± 0.3 ms for control *n* = 7 cells, *p* < 0.05.

The Wnt signaling is evolutionally conserved and is a critical regulator of gene expression (Cadigan & Nusse, 1997; Liang et al., 2020). In the canonical Wnt pathway (Figure 1A), Wnt receptor activation leads to inhibition of GSK‐3β, a key mediator of β‐catenin degradation; this causes cytosolic accumulation of β‐catenin, which then translocates into the nucleus for regulation of target gene transcription. Increased activity of the Wnt/β‐catenin pathway has been found in arrhythmogenic heart disease, such as myocardial infarction and heart failure (Dawson et al., 2013; Hou et al., 2016; Malekar et al., 2010). We and others have previously demonstrated that Wnt/β‐catenin signaling reduces Na^+^ channel transcript (*Scn5a*), protein (Na_v_1.5), and current (*I*_Na_) in neonatal rat ventricular myocytes (NRVMs) (Liang et al., 2015; Lu et al., 2020) and in HL‐1 cells (Wang et al., 2016; Zhao et al., 2019). Consistent with a selective effect on *I*_Na_, the L‐type Ca^2+^ current was not affected by Wnt/β‐catenin signaling in NRVMs (Liang et al., 2015). In addition, cardiac activation of Wnt/β‐catenin signaling at early embryonic stage reduced Na_v_1.5 protein in adult mouse hearts (Gillers et al., 2015; Li et al., 2018). However, because of species differences between rodent and human hearts, it is not known if Wnt/β‐catenin signaling regulates Na_v_1.5 in human cardiomyocytes and if inhibition of Wnt/β‐catenin signaling can rescue the reduced Na_v_1.5 in patient cardiomyocytes.

In this study, we demonstrate that activation of Wnt/β‐catenin signaling in healthy human iPSC‐derived cardiomyocytes (iPSC‐CMs) reduces *SCN5A* mRNA and Na_v_1.5 protein leading to reductions in *I*_Na_ density and alterations in *I*_Na_ gating kinetics. Moreover, upregulation of Na_v_1.5 was achieved in BrS iPSC‐CMs after inhibition of the Wnt/β‐catenin signaling by two different strategies: a small‐molecule inhibitor and shRNA‐mediated β‐catenin knockdown. Importantly, β‐catenin knockdown restored the peak *I*_Na_ density in BrS iPSC‐CMs to near (78% of) that in healthy iPSC‐CMs.

## 2 METHODS

The generation of human iPSC lines were completed in Dr. Joseph C. Wu's lab at Stanford Cardiovascular Institute with Institutional Ethics Committee approved protocols (IRB 29904 and SCRO 485) and informed written consents were given prior to the inclusion of subjects in the study. Investigations involving human cells conformed to the principles outlined in the Declaration of Helsinki and were approved by the institutional review committee at the University of Ottawa Heart Institute.

### 2.1 Human‐induced pluripotent stem cells (iPSCs) and iPSC‐derived cardiomyocytes (iPSC‐CMs)

The generation of iPSCs from two BrS patients has been described previously (Liang et al., 2016). The first patient (male, 44 years old, iPSC ID: SCVI128‐C2, designated as BrS Line 1 in this study) had unstable ventricular tachycardia and ECG revealed a pattern characteristic of BrS. This patient contained a benign variant (R620H) and a disease‐causing variant (R811H) in each of the two *SCN5A* alleles (Calloe et al., 2013; Liang et al., 2016). The second BrS patient (male, 53 years old, iPSC ID: SCVI129‐C1, designated as BrS Line 2) harbored a 1‐base pair deletion mutation (causing a frame shift mutation, 4190ΔA) in one of the two *SCN5A* alleles, which is anticipated to generate a truncated non‐functional Na_v_1.5 (Liang et al., 2016). The healthy human iPSC line (ID: SCVI273, designated as Healthy Line 2) was generated in Dr. J.C. Wu's lab from a healthy volunteer (female, 41 years old) as described (Kitani et al., 2019).

iPSC lines were maintained and differentiated into cardiomyocytes in a chemically defined medium according to standard operating procedures (SOPs) established in Dr. J.C. Wu's lab (Burridge et al., 2014). Briefly, iPSCs (passage 15–40) at 80%–90% confluency were treated with 6 μM CHIR‐99021 (Selleck Chemicals, S1263) from Day 0 to 2 in CDM3 medium, which was composed of RPMI 1640 (Corning, 10‐040‐CM), 213 μg/mL L‐ascorbic acid 2‐phosphate (Wako Chemicals, 321‐44823), and 500 μg/mL recombinant human albumin (ScienCell, OsrHSA‐100). Cells were maintained from Day 2 to 4 in CDM3 medium containing 4 μM Wnt‐C59 (Selleck Chemicals, S7037). At Day 4, cells were cultured in CDM3 medium (without any additional factors) and spontaneously beating regions were generally observed starting Day 7–10 under a microscope. Cells were glucose‐starved from Day 10 to 14 to purify cardiomyocytes. Glucose‐free CMD3 medium was prepared by replacing glucose with 4 mM L‐lactic acid (Wako Chemicals, 129‐02666). To minimize well‐to‐well variations during the differentiation, cells at Day 14–20 were lifted with TrypLE (Life Technologies, 12605‐036), pooled, and replated into new plates at a density of 250,000 cells/cm^2^ in RPMI 1640 medium (Corning, 10‐040‐CM) containing 1x B‐27 supplement (ThermoFisher, 17504044). Cells at Day 30–45 of differentiation were used in this study. An additional line of healthy iPSC‐CMs (designated as Healthy Line 1) was purchased from Axolbio (Cambridge, UK, Catalogue No.: ax2505). iPSCs were derived from cord blood CD34^+^ cells collected from a healthy male newborn by reprogramming with episomal vectors expressing the reprogramming factors. Cardiac differentiation was performed in iPSC monolayer culture in a chemically defined medium.

### 2.2 Activation and suppression of Wnt/β‐catenin signaling in iPSC‐CMs

To activate Wnt signaling, iPSC‐CMs were treated with 5 μM CHIR‐99021 (Selleck Chemicals, S1263, stock solution was made with DMSO at 10 or 100 mM) for 48 h before RNA extraction, qRT‐PCR, and patch‐clamp studies as described below. Control cells were cultured in medium containing equal volume of DMSO (Sigma‐Aldrich, D2650). To suppress Wnt signaling, BrS iPSC‐CMs were transduced with adenovirus expressing an shRNA targeting human *CTNNB1* encoding β‐catenin (Ad‐shRNA‐β‐catenin, Vector Biolabs, shADV‐206246) or control adenovirus expressing a non‐silencing shRNA (Ad‐shRNA‐control, Vector Biolabs, 1781) at a MOI of 10. At Day 5–8 after virus transduction, cells were used for analyses (qPCR, western blot, and patch‐clamp). In another group of studies, Wnt signaling was inhibited in BrS iPSC‐CMs by including Wnt‐C59 (4 μM, Selleck Chemicals, S7037) in culture medium for 3 days. Control cells were cultured in medium containing equal volume of DMSO (Sigma‐Aldrich, D2650).

### 2.3 Real‐Time quantitative PCR

Total RNA was isolated from iPSC‐CMs with a RNeasy mini kit (Qiagen, 74104) and cDNA was synthesized with a High‐Capacity cDNA Reverse Transcription Kit (Applied Biosystems, 4368814). Real‐time quantitative PCR was performed on a CFX Connect Real‐Time PCR Detection System (Bio‐Rad) using iTaq Universal SYBR Green Supermix (Bio‐Rad, 1725121). Information on the qPCR primers was included in Table 1. The level of target gene transcripts was normalized to the level of housekeeping gene transcript (*HPRT1* or the mean values of *HPRT1*, *RPL32*, and *PPIA*) in the same sample. Some qPCR experiments were performed with TaqMan Gene Expression Assays (Life Technologies, Assay IDs: Hs00165693 for *SCN5A*, and Hs02800695 for *HPRT1*). Results were analyzed with the 2^−ΔΔ C(t)^ method.

**TABLE 1:** Primers for SYBR green qPCR.

Genes	Forward (5′ — 3′)	Reverse (5′ — 3′)	Amplicon size (bp)	Target exon(s)
*SCN5A*	ATCCCAGGGCTGAAGACCAT	AAGACGCTGAGGCAGAAGAC	95	6–7
*TBX3*	CATGGAGCCCGAAGAAGAGG	AACATTCGCCTTCCCGACTT	123	1–2
*KCNJ2*	GGTTTGCTTTGGCTCACTCG	GAACATGTCCTGTTGCTGGC	111	1–2
*CACNA1C*	AGATGACTGCTTATGGGGCT	GATTGCACTGGACTGGATGC	122	21–22
*CTNNB1*	GAAGCTGGTGGAATGCAAGC	AGTCCCAAGGAGACCTTCCA	138	7–9
*AXIN2*	TAACCCCTCAGAGCGATGGA	CCTCCTCTCTTTTACAGCAGGG	80	1–2
*LEF1*	CCCGTGAAGAGCAGGCTAAA	TCTTGGACCTGTACCTGATGC	158	9–11
*HPRT1*	CCTGGCGTCGTGATTAGTGA	CGAGCAAGACGTTCAGTCCT	137	1–2
*RPL32*	GTTACGACCCATCAGCCCTTG	CATGATGCCGAGAAGGAGATGG	93	1–2
*PPIA*	ACGTGGTATAAAAGGGGCGG	CCAGGCCCCTTACCTCAAAG	163	1–2

### 2.4 Immunocytostaining

iPSC‐CMs cultured on 8‐chamber culture slides (ThermoFisher, 154461PK) were fixed with 100% methanol at −20°C for 10 min. Cells were blocked and permeabilized in Dako protein block (Agilent, X0909) containing 0.1% (w/v) saponin (Sigma‐Aldrich, 47036) at room temperature for 90 min. Cells were then incubated with primary antibodies (see below) diluted in the same blocking solution at 4°C for overnight. Then cells were washed with phosphate‐buffered saline (PBS) and incubated with secondary antibodies (see below) at room temperature for 1 h. Cells were washed with PBS and mounted with ProLong gold antifade reagent containing DAPI (ThermoFisher, P36931). Primary antibodies used were monoclonal rabbit anti‐Na_v_1.5 antibody targeting the C‐terminus (1:100, Cell Signaling, 14421), monoclonal mouse anti‐α‐sarcomeric actinin antibody (1:400, Sigma, A7811), rabbit anti‐β‐catenin antibody (1:100, Cell Signaling, 8480), and mouse anti‐N‐Cadherin antibody (BD Bioscience, 610920). Secondary antibodies used were goat anti‐mouse IgG Alexa Fluor‐568 (1:300, ThermoFisher, A‐11004) and anti‐rabbit IgG Alexa Fluor‐488 (1:300, ThermoFisher, A‐11008). Fluorescent cellular images were taken with a ZEISS confocal microscope equipped with the Airyscan technique for high‐resolution imaging (Zeiss Elyra S.1 LSM 880).

### 2.5 Western blotting

iPSC‐CMs were homogenized in RIPA buffer (ThermoFisher, 89900) supplemented with Halt protease inhibitor cocktail (ThermoFisher, 78430). Protein concentration was determined by BCA assay (Redinbaugh & Turley, 1986) using Pierce Rapid Gold BCA Protein Assay Kit (ThermoFisher, A53227). Cell lysates (30 μg protein per lane) were run on a 4%–12% SDS‐polyacrylamide gel (ThermoFisher, NW04120BOX) and transferred onto a PVDF membrane. The Novex™ Sharp Pre‐stained protein standard (ThermoFisher, LC5800) was used as a molecular weight marker. The transferred membrane was incubated with a primary antibody overnight at 4°C, followed by a 2‐h incubation with a peroxidase‐conjugated anti‐rabbit (ThermoFisher, 31460) or anti‐mouse (KPL, 04‐18‐15) secondary antibody at 1:2000 dilution. Primary antibodies used were: polyclonal rabbit anti‐Na_v_1.5 antibody that binds to the intracellular loop between domains I and II (1:1000, a kind gift from Dr. Hugues Abriel, University of Bern, Switzerland) or monoclonal rabbit anti‐Na_v_1.5 antibody targeting the C‐terminus (1:1000, Cell Signaling, 14421), rabbit anti‐Ca_v_1.2 antibody (1:1000, Alomone Labs, ACC‐003), rabbit anti‐β‐catenin antibody (1:1000, Cell Signaling, 8480), mouse anti‐α‐sarcomeric actinin antibody (α‐SA, 1:1000, Sigma, A7811), rabbit anti‐K_ir_2.1 antibody (1:1000, Abcam, ab65796), and rabbit anti‐Tbx3 antibody (1:1000, ThermoFisher, 42–4800). Immunoreactivity was detected using SuperSignal West Pico PLUS Chemiluminescent Substrate (ThermoFisher, 34580). Equal protein loading of the gels was assessed by re‐probing the membranes with rabbit anti‐calnexin antibody (1:2000, Abcam, ab22595). Band densities in western blot experiments were quantified using the “Measurements” function within ImageJ (http://www.yorku.ca/yisheng/Internal/Protocols/ImageJ.pdf) or the “Gel Analyzer Protocol” function of ImageJ (https://imagej.nih.gov/ij/docs/menus/analyze.html#gels; results calculated with these two methods were found to be not different) and presented in the manuscript after normalization to calnexin band densities in the same membranes.

### 2.6 Electrophysiology

Electrophysiology experiments were carried out using standard whole‐cell patch‐clamp technique (Liang et al., 2014; Liang et al., 2015) with an AxoPatch 200B amplifier (Molecular Devices) at a sampling rate of 20 kHz and low‐pass Bessel‐filtered at 5 kHz. Pipettes were pulled from borosilicate glass tubes (OD:1.50 mm, ID: 1.17 mm, with filament, Warner Instruments, G150TF‐3) using a temperature‐controlled pipette puller (HEKA, PIP6). Pipettes had tip resistances of 2–5 MΩ when filled with an internal pipette solution containing (in mM): NaCl 5, CsF 125, EGTA 10, HEPES 10, and Mg‐ATP 5 (pH = 7.2 with CsOH). iPSC‐CMs were placed in a perfusion chamber (Warner Instruments, RC‐22) on the stage of an inverted microscope (Olympus, IX‐50). Voltage‐gated Na^+^ currents were recorded in voltage‐clamp mode at room temperature with cells perfused in a bath solution containing (in mM) NaCl 20, TEA‐Cl 50, CsCl 67, MgCl_2_ 1, CaCl_2_ 1, glucose 10, and HEPES 10 (pH = 7.4 with CsOH) (*E*_Na_ = +35.3 mV). In some studies, NaCl in the bath solution was increased to 100 mM (*E*_Na_ = +75.7 mV) with reductions in TEA‐Cl and CsCl to keep the osmolarity constant. Either 0.1 mM CdCl_2_ or 10 μM nifedipine was included in the bath solution to inhibit L‐type Ca^2+^ current during the whole‐cell recording. Series resistance was compensated by 70%–80%. Cells were held at −120 mV and *I*_Na_ was elicited by a family of 50‐ms (or 200‐ms) voltage steps to potentials ranging from −90 to +30 mV (when 20 mM Na^+^ bath solution was used) or from −90 to +70 mV with 5‐mV increments and a cycle length of 600 ms. In some recordings, cells were held at −90 mV and stepped to −120 mV for 200 ms before the voltages steps to elicit *I*_Na_ (Zhang et al., 2018). *I*_Na_ conductance and gating kinetics were measured using protocols described in figure legends.

### 2.7 Statistical analysis

Data are expressed as mean ± standard deviation (SD) with *p* < 0.05 considered significant. Information on sample numbers, including the number of cells or samples (*n*) and the number of cell differentiations (*N*), is included in the figure legends. Differences between two means were evaluated by two‐tailed Student's *t*‐test. Differences among multiple means were assessed by one‐way analysis of variance (ANOVA). When significance was detected by ANOVA, differences among individual means were evaluated post hoc by Bonferroni's test.

## 3 RESULTS

### 3.1 Wnt/β‐catenin signaling reduces *SCN5A* mRNA, Na_v_1.5 protein and *I*_Na_ in male iPSC‐derived cardiomyocytes

Activation of Wnt/β‐catenin signaling was induced in male healthy iPSC‐CMs (Healthy Line 1) by treatment with CHIR‐99021 (CHIR) for 48 h. CHIR is a cell‐permeant small‐molecule inhibitor of GSK‐3β and is a commonly used Wnt/β‐catenin pathway activator (Figure 1A). The increases in *AXIN2* mRNA (Figure 1B), a known target of the Wnt/β‐catenin pathway (Jho et al., 2002; Liang et al., 2015), suggests successful activation of the pathway by CHIR treatment. In the same CHIR‐treated samples, the transcript of *SCN5A*, encoding Na_v_1.5, was reduced (Figure 1B). Results from three different experiments were summarized in Figure 1B. The smaller changes in *AXIN2 and SCN5A* mRNA in experiment #1 are likely due to the reduced activity of the CHIR molecules after prolonged storage (>6 months) in stock solution. Therefore, CHIR stock solutions that were prepared within 3 months were used in subsequent studies to reduce variations among the experiments. Regardless, the changes in *SCN5A* and *AXIN2* mRNA were highly correlated (*p* = 0.0021, Figure 1B). This suggests that the reductions in *SCN5A* mRNA in CHIR‐treated cells are a direct effect of Wnt/β‐catenin signaling activation.

Western blot revealed two distinct bands for Na_v_1.5 in iPSC‐CMs with one band near 260 kDa and another band with a higher molecular weight (Figure 1C, arrowhead). CHIR treatment for 48 h led to a 93% reduction (*p* < 0.0001) in higher band and a 63% reduction (*p* = 0.0035) in lower band (Figure 1C), as well as a 77% reduction in the higher‐to‐lower band ratio (*p* < 0.001), and an 80% reduction in total Na_v_1.5 (*p* = 0.0002). To investigate if reduced Na_v_1.5 protein is associated with changes in voltage‐gated Na^+^ current (*I*_Na_), whole‐cell patch‐clamp recording was performed in two different ionic conditions in male healthy iPSC‐CMs. In the first experiment, *I*_Na_ was recorded with a bath solution (Liang et al., 2015) containing 20 mM Na^+^ (*E*_Na_ = +35 mV) and 0.1 mM Cd^2+^ (to inhibit L‐type Ca^2+^ current in whole‐cell recording). CHIR treatment led to an 82% reduction (*p* < 0.05) in peak *I*_Na_ density (−10.5 ± 8.3 pA/pF at −20 mV, *n* = 9 cells, vs. control −58.0 ± 53.14 pA/pF, *n* = 6 cells, Figure 1D). In the second experiment, the bath solution contained 100 mM Na^+^ (*E*_Na_ = +76 mV) without Cd^2+^ (instead, nifedipine was included to block L‐type Ca^2+^ current). CHIR treatment led to an 84% reduction in peak *I*_Na_ density (−22.3 ± 7.9 pA/pF at −20 mV, *n* = 7 cells vs. control −138.3 ± 20.0 pA/pF, *n* = 12 cells, Figure 1E). Accordingly, *I*_Na_ conductance was reduced by 76% after CHIR treatment (Figure 1Ec). In both experiments (Figure 1D,E), the apparent reversal potentials of the currents were consistent with the calculated equilibrium potentials of Na^+^ (*E*_Na_), suggesting that the recorded current was primarily carried by Na^+^.

The reduced *I*_Na_ density in CHIR‐treated iPSC‐CMs is consistent with our previous study (Liang et al., 2015) showing reduced *I*_Na_ density in rat cardiomyocytes after CHIR or Wnt3a treatment. However, in contrast to observations in rat cardiomyocytes in which *I*_Na_ gating kinetics were not affected (Liang et al., 2015), *I*_Na_ gating kinetics were altered by CHIR treatment in iPSC‐CMs: the half‐maximal activation voltage (*V*_1/2_) was increased by 13.9 mV (−20.1 ± 0.4 mV in CHIR group, *n* = 7 cells vs. −34.0 ± 0.3 mV in control, *n* = 8 cells, Figure 1Ed) and *k* values (slope factor) were also increased (7.5 ± 0.3 vs. control 4.8 ± 0.1, *p* < 0.01). In addition, the steady‐state inactivation curve of *I*_Na_ showed a small (4.8 mV) shift to the left (*V*_1/2 =_ − 81.2 ± 0.4 mV, *n* = 5 cells vs. control −76.4 ± 0.2 mV, *n* = 14 cells, *p* < 0.01, Figure 1Ee). Recovery of *I*_Na_ from inactivation was delayed (*p* < 0.05) by CHIR (time for 50% recovery, *t* = 16.6 ± 1.6 ms, *n* = 5 cells, vs. control 8.7 ± 0.3 ms, *n* = 7 cells, Figure 1Ef). These CHIR‐induced alterations in *I*_Na_ kinetics will reduce the *I*_Na_ amplitude during an action potential, which is known to be proarrhythmic in the heart.

### 3.2 Wnt/β‐catenin signaling reduces *SCN5A* mRNA and Na_v_1.5 protein in female iPSC‐derived cardiomyocytes

**FIGURE 2:**
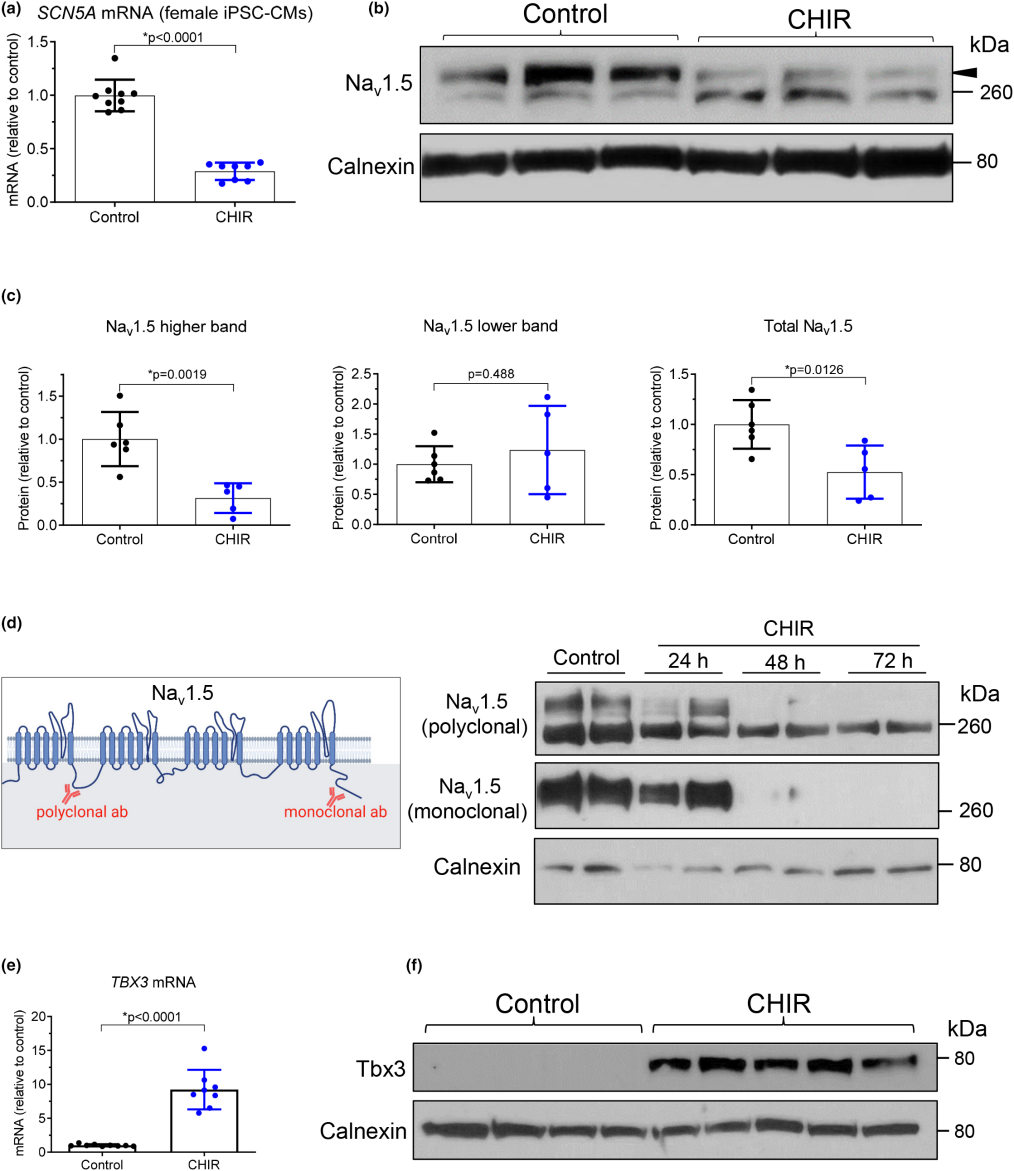
Wnt/β‐catenin signaling reduces *SCN5A* mRNA and Na_v_1.5 protein in female iPSC‐derived cardiomyocytes. (a) qRT‐PCR showing reduced *SCN5A* mRNA in female iPSC‐CMs after treatment with CHIR for 48 h (5 μM, *n* = 8 samples from two cell differentiations). Control group was maintained in culture medium containing equal amount of DMSO (*n* = 9 samples from two cell differentiations). Data were analyzed by two‐tailed *t*‐test. (b) Representative western blot showing reduced Na_v_1.5 (using a polyclonal antibody) in female iPSC‐CMs after treatment with CHIR or DMSO (control) for 48 h. Two Na_v_1.5 bands were observed with one near 260 kDa and another with a higher molecular weight (arrowhead). (c) Quantification of Na_v_1.5 band densities in panel B (normalized to calnexin), showing reduced Na_v_1.5 higher band in CHIR group (*n* = 5 samples from two cell differentiations) as compared to control, DMSO‐treated group (*n* = 6 samples from two cell differentiations). Data were analyzed by two‐tailed *t*‐test. (d) Left panel, diagram showing the binding sites of the two anti‐Na_v_1.5 antibodies. The polyclonal antibody binds to the intracellular loop between domains I and II and the monoclonal antibody binds to the C‐terminus of Na_v_1.5. Right panel, western blot showing time‐dependent reductions in Na_v_1.5 in female iPSC‐CMs after treatment with CHIR (5 μM) for 24, 48, or 72 h. The same samples were probed sequentially with two different anti‐Na_v_1.5 antibodies shown in left panel. The lower band (near 260 kDa) was not detected by the monoclonal antibody. (e) qRT‐PCR showing CHIR‐induced upregulation of *TBX3* mRNA, a known repressor of *Scn5a*/Na_v_1.5 expression, in female iPSC‐CMs (*n* = 8 samples from three cell differentiations) after CHIR treatment for 48 h. Control group was maintained in culture medium containing equal amount of DMSO. *p < 0.0001 by two‐tailed *t*‐test. (f) Western blot showing increased Tbx3 protein in female iPSC‐CMs after treatment with CHIR for 48 h.

To investigate if the inhibition of Na_v_1.5 by Wnt/β‐catenin signaling is reproduced in a different iPSC line and if this effect is dependent on the sex of the cells, the effects of CHIR were further studied in iPSC‐CMs derived from a healthy female volunteer. CHIR treatment of female iPSC‐CMs induced a 71% reduction in *SCN5A* mRNA (0.29 ± 0.08, *n* = 8, vs. control 1.0 ± 0.15, *n* = 9, Figure 2a) and a 69% reduction in the higher Na_v_1.5 band (0.31 ± 0.17, *n* = 5, vs. control 1.0 ± 0.31, *n* = 6, Figure 2b,c). However, the lower Na_v_1.5 band was not affected (*p* = 0.488) by CHIR treatment in female iPSC‐CMs. Regardless, total Na_v_1.5 was consistently reduced by CHIR in both male and female iPSC‐CMs. The higher and lower bands for Na_v_1.5 were further investigated by probing the same western blot samples of female iPSC‐CMs with two different anti‐Na_v_1.5 antibodies: a monoclonal antibody that binds to the C‐terminus of the Na_v_1.5 protein, and a polyclonal antibody that binds to the intracellular loop between domains I and II (Figure 2d, left panel). As shown in Figure 2d (right panel), only the higher band was detected by both antibodies.

We have previously identified two mechanisms that underlie the inhibition of *Scn5a*/Na_v_1.5 by Wnt/β‐catenin signaling in rat cardiomyocytes (Lu et al., 2020). The first mechanism is direct repression of *Scn5a* gene expression by the binding of TCF4 (the downstream effector of β‐catenin) to *Scn5a* promoter via two sites that are highly conserved among species including human and rats (Lu et al., 2020). The second mechanism is Wnt signaling‐induced upregulation of Tbx3, a potent repressor of *Scn5a*/Na_v_1.5/*I*_Na_ (Lu et al., 2020). In female healthy iPSC‐CMs, CHIR treatment increased in both *TBX3* mRNA and protein (Figure 2e,f), consistent with our previous observations in rat cardiomyocytes (Lu et al., 2020), suggesting that the upregulation of Tbx3 by Wnt/β‐catenin signaling is conserved in both rat and human cardiomyocytes.

### 3.3 A small‐molecule inhibitor of Wnt signaling upregulates Na_v_1.5 protein in Brugada syndrome iPSC‐CMs

**FIGURE 3:**
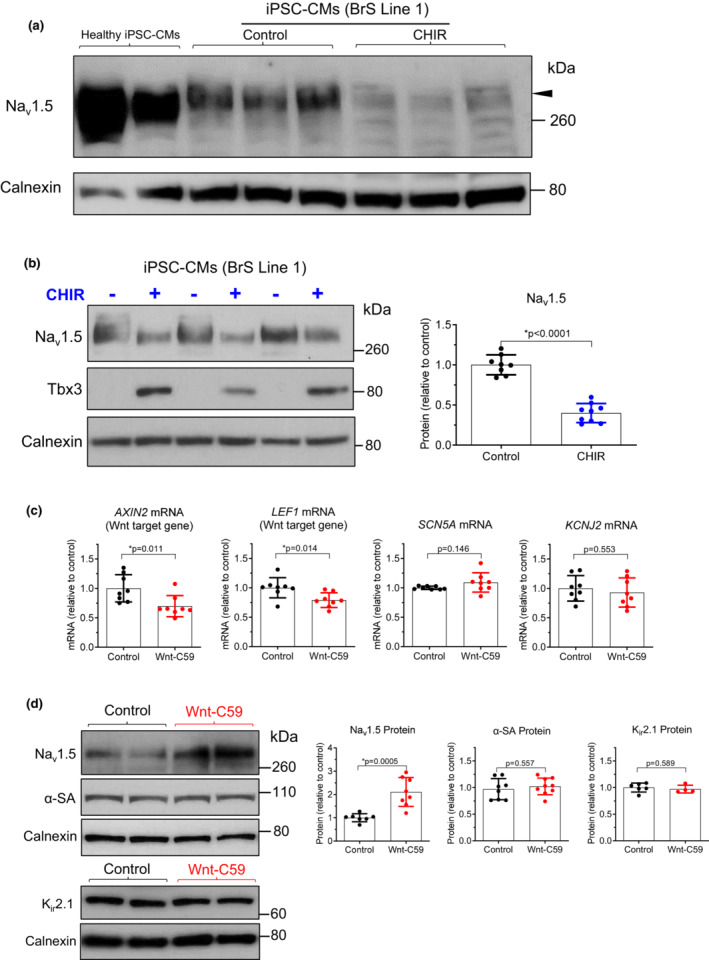
Regulation of Na_v_1.5 protein by Wnt/β‐catenin signaling in Brugada syndrome (BrS) iPSC‐CMs. (a) Representative western blot showing reduced Na_v_1.5 (polyclonal antibody) in BrS iPSC‐CMs after treatment with CHIR (right three lanes) for 48 h, as compared to DMSO‐treated cells (control, middle three lanes). Two samples from healthy iPSC‐CMs (female) were included in the first two lanes for side‐to‐side comparison which showed a lower level of Na_v_1.5 in BrS iPSC‐CMs. This Brugada syndrome (BrS) patient had a benign variant (R620H) and a disease‐causing variant (R811H) on each of the two *SCN5A* alleles. (b) Left, western blot showing upregulation of Tbx3 and downregulation of Na_v_1.5 (monoclonal antibody) in BrS iPSC‐CMs after CHIR treatment. Right, quantification of Na_v_1.5 band densities (normalized to calnexin) showing reduced Na_v_1.5 in BrS iPSC‐CMs after treatment with CHIR (*n* = 9 samples from three cell differentiations) as compared to control, DMSO‐treated cells (*n* = 8 samples from three cell differentiations). Data were analyzed by two‐tailed *t*‐test. (c) qRT‐PCR showing reduced *AXIN2* and *LEF1* mRNA, two known targets of Wnt/β‐catenin signaling, in BrS iPSC‐CMs after treatment with Wnt‐C59, a small‐molecule inhibitor of Wnt/β‐catenin signaling. *SCN5A* and *KCNJ2* mRNA were not affected by Wnt‐C59 treatment. *n* = 8 samples from two different differentiations for both control (DMSO) and CHIR groups. Data were analyzed by two‐tailed *t*‐test. (d) Western blot showing upregulation of Na_v_1.5 (monoclonal antibody) in BrS iPSC‐CMs after Wnt‐C59 treatment (*n* = 8 samples from two differentiations of cells) as compared to control, DMSO‐treated cells (*n* = 7 samples from two differentiations). Myocyte maturity markers, α‐sarcomeric actinin (α‐SA, *n* = 8–9 samples from two differentiations) and K_ir_2.1 (*n* = 4–5 samples from two differentiations), were not affected by Wnt‐C59 treatment. Data were analyzed by two‐tailed *t*‐test.

The inhibition of Na_v_1.5 channels by Wnt/β‐catenin signaling in both human and rat (Liang et al., 2015) cardiomyocytes suggests that it is a conserved mechanism. To explore the translational potential of these findings, we used BrS cardiomyocytes as an example to investigate if inhibition of Wnt/β‐catenin signaling increases Na_v_1.5 in human arrhythmogenic heart disease. We first used iPSC‐CMs derived from a BrS patient (BrS Line 1) who had unstable ventricular tachycardia and two missense variants in the *SCN5A* gene (R620H and R811H on each of the two alleles) were identified in this patient (Liang et al., 2016). Heterologous expression studies (Calloe et al., 2013) showed that the R620H variant is benign, but the R811H variant reduced *I*_Na_ by ~50%, although it was not clear if the reduction in current is caused by altered channel properties or by reduced Na_v_1.5 expression. The study by Liang et al. (2016) also demonstrated that these BrS iPSC‐CMs had reduced *I*_Na_ as compared to healthy iPSC‐CMs. In agreement with these previous findings, the present study showed that these BrS iPSC‐CMs had a lower Na_v_1.5 level than healthy iPSC‐CMs (Figure 3a). CHIR treatment of BrS iPSC‐CMs further reduced Na_v_1.5 by 60% (0.40 ± 0.12, *n* = 9, vs. control 1.00 ± 0.12, *n* = 8, *p* < 0.0001, Figure 3a,b). This suggests that the inhibition of Na_v_1.5 by Wnt/β‐catenin signaling is found in both healthy and BrS cardiomyocytes. In addition, CHIR treatment also upregulated Tbx3 protein in BrS iPSC‐CMs (Figure 3b), which is consistent with observations in healthy iPSC‐CMs.

To investigate if blocking Wnt/β‐catenin signaling upregulates Na_v_1.5 channel expression in BrS iPSC‐CMs, we first tested a small‐molecule inhibitor of Wnt signaling, Wnt‐C59, which inhibits the palmitoylation and secretion of Wnt proteins (Proffitt et al., 2013). The transcripts of *AXIN2* and *LEF1*, two genes known to be regulated by Wnt/β‐catenin signaling in cardiomyocytes (Liang et al., 2015), were reduced by 30% and 21%, respectively (Figure 3c), suggesting inhibition of Wnt/β‐catenin signaling in BrS iPSC‐CMs after Wnt‐C59 treatment. Na_v_1.5 protein was increased by 2.1‐fold after Wnt‐C59 treatment (2.11 ± 0.62, *n* = 8, vs. control 1.00 ± 0.17, *n* = 7, *p* = 0.0005, Figure 3d). However, the *SCN5A* mRNA was not affected by Wnt‐C59 (1.09 ± 0.16, *n* = 8, vs. control 1.00 ± 0.03, *n* = 8, *p* = 0.146, Figure 3c). This suggests that the upregulation of Na_v_1.5 after Wnt signaling inhibition is likely mediated by posttranscriptional mechanisms.

Considering the role of Wnt/β‐catenin signaling in cardiac differentiation (Liang et al., 2020), we investigated if Wnt‐C59 altered the maturation of BrS iPSC‐CMs, which may explain the observed upregulation of Na_v_1.5 in these cells. It has been demonstrated that the protein level of α‐sarcomeric actinin (α‐SA, α‐actinin 2) is associated with the maturation degree of iPSC‐CMs (Cai et al., 2019). In addition, upregulation of *KCNJ2* mRNA, encoding K_ir_2.1 and *I*_K1_, has been demonstrated in more mature iPSC‐CMs (Feyen et al., 2020). In the present study, none of these maturation‐associated factors (α‐SA protein, *KCNJ2* mRNA or K_ir_2.1 protein) was affected by Wnt‐C59 treatment (Figure 3c,d), suggesting that Wnt‐C59 treatment did not significantly impact the maturation of BrS iPSC‐CMs.

### 3.4 β‐catenin knockdown upregulates Na_v_1.5 protein in Brugada syndrome iPSC‐CMs

**FIGURE 4:**
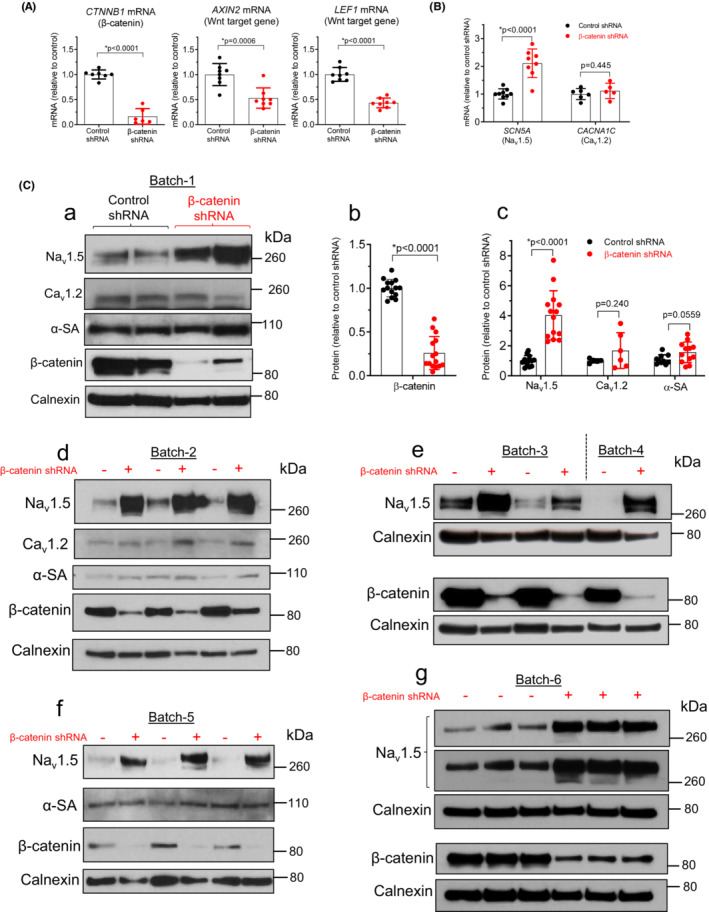
β‐catenin knockdown increases *SCN5A* mRNA and Na_v_1.5 protein in Brugada syndrome iPSC‐CMs. (A) qRT‐PCR showing reduced *CTNNB1* mRNA (encoding β‐catenin) in BrS iPSC‐CMs expressing β‐catenin shRNA (*n* = 6 samples) as compared to cells expressing a control, non‐silencing shRNA (*n* = 7 samples). *AXIN2* and *LEF1* mRNA, two known targets of Wnt/β‐catenin signaling, were reduced after β‐catenin knockdown in BrS iPSC‐CMs (*n* = 8 samples for both groups). Cells were from two iPSC differentiations. Data were analyzed by two‐tailed *t*‐test. (B) qRT‐PCR showing increased *SCN5A* mRNA (*n* = 9 samples for control‐shRNA; *n* = 8 samples for β‐catenin‐shRNA) in BrS iPSC‐CMs expressing β‐catenin shRNA. mRNA of *CACNA1C* was not affected by β‐catenin shRNA. Cells were from two to three iPSC differentiations. Data were analyzed by two‐tailed *t*‐test. (C) Western blot showing reduced β‐catenin (*n* = 15 samples) and increased Na_v_1.5 (*n* = 14 samples) in BrS iPSC‐CMs expressing β‐catenin shRNA as compared to control shRNA group. Calnexin was used as a loading control. Cells from a total of six batches of iPSC differentiation were used for this Na_v_1.5 western blot study (a polyclonal antibody for panels Ce and Cg, and a monoclonal antibody for panels Ca, Cd, and Cf). Panels Cd to Cg: the β‐catenin shRNA groups were labeled as “+”, and the control‐shRNA groups were labeled as “−”. Batch‐1 contained three β‐catenin shRNA samples with only two shown in the images. The two samples in Batch‐4 were not included in the Na_v_1.5 summary (panel Cc) because the control sample did not show a clear band. In panel Cg, two blots for Na_v_1.5 with different exposure times are shown. Ca_v_1.2 and α‐sarcomeric actinin (α‐SA) only showed small increases in one batch of the cells (Batch‐2). Panels Cb and Cc show summary data and were analyzed by two‐tailed *t*‐test.

To further investigate the regulation of Na_v_1.5 by Wnt signaling inhibition, we used a different strategy to block Wnt/β‐catenin signaling in BrS iPSC‐CMs: β‐catenin, the intracellular mediator of the signaling pathway, was knocked down by expressing an shRNA targeting the *CTNNB1* gene (encoding β‐catenin). Successful knockdown was confirmed by an 84% reduction in β‐catenin mRNA (0.16 ± 0.16 *n* = 6 vs. a non‐silencing control shRNA 1.0 ± 0.09 *n* = 7, Figure 4A) and a 74% reduction in β‐catenin protein (0.26 ± 0.19 *n* = 15 vs. control shRNA 1.00 ± 0.10 *n* = 14, Figure 4Cb). Accordingly, the transcripts of *AXIN2* and *LEF1*, two target genes of Wnt*/*β‐catenin signaling, were reduced by 46% and 56%, respectively (Figure 4A). As compared with Wnt‐C59 (Figure 3c), β‐catenin knockdown induced greater reductions in *AXIN2* and *LEF1* mRNA, suggesting a higher degree of Wnt signaling inhibition. shRNA‐mediated knockdown of β‐catenin increased *SCN5A* mRNA by 2.1‐fold (2.11 ± 0.52, *n* = 8 vs. control shRNA 1.01 ± 0.18, *n* = 9, *p* < 0.0001, Figure 4B), but the L‐type Ca^2+^ channel gene *CACNA1C* was not affected (*p* = 0.445, Figure 4B).

Western blot analyses of BrS iPSC‐CMs from six different batches of iPSC differentiation consistently showed Na_v_1.5 upregulation after β‐catenin knockdown (Figure 4C). On average, Na_v_1.5 protein was increased by 4.0‐fold (4.04 ± 1.63, *n* = 14, vs. control shRNA 1.01 ± 0.34, *n* = 13, *p* < 0.0001 Figure 4Cc). The protein levels of α‐SA and Ca_v_1.2 (the α subunit of L‐type Ca^2+^ channel) showed small increases only in one batch of the cells (Batch‐2) after β‐catenin knockdown (Figure 4Cd), suggesting possible changes in maturation of the Batch‐2 cells. However, the upregulation of Na_v_1.5 was observed in all the six batches of cells tested. These observations suggest that the upregulation of Na_v_1.5 after β‐catenin knockdown in BrS iPSC‐CMs is a direct effect of Wnt/β‐catenin signaling inhibition, rather than an indirect effect secondary to alterations in cell maturation.

### 3.5 β‐catenin knockdown increases cell surface Na_v_1.5 and *I*_Na_ in Brugada syndrome iPSC‐CMs

**FIGURE 5:**
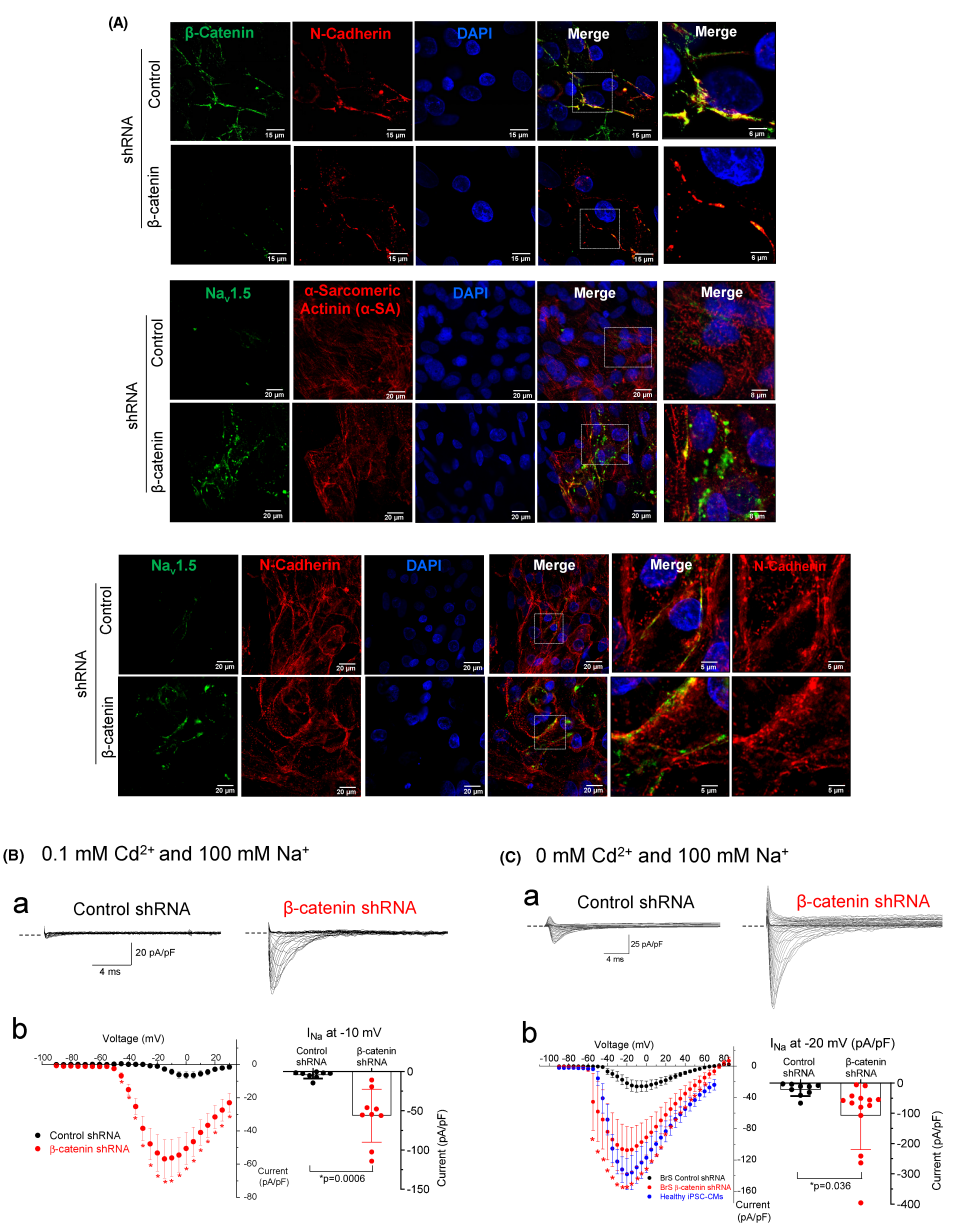
β‐catenin knockdown increases cell surface Na_v_1.5 and *I*_Na_ in BrS iPSC‐CMs. (A) Representative confocal images of BrS iPSC‐CMs showing reduced β‐catenin and increased Na_v_1.5 in β‐catenin shRNA group. Cells were co‐stained with either α‐sarcomeric actinin (α‐SA, a marker of cardiomyocytes) or N‐Cadherin (a plasma membrane protein expressed in cardiomyocytes). The fifth column shows the expanded view of the white box in the fourth column. In the bottom panel, N‐Cadherin was shown in the sixth column to show co‐localization between N‐Cadherin and Na_v_1.5 (the fifth column). (B) *I*_Na_ was recorded from BrS iPSC‐CMs with a bath solution containing 100 mM Na^+^ (*E*_Na_ = +76 mV) and 0.1 mM Cd^2+^ (to inhibit L‐type Ca^2+^ current in whole‐cell recording). (a) Representative *I*_Na_ recorded from BrS iPSC‐CMs with adenoviral expression of a control non‐silencing shRNA (left) or a shRNA targeting β‐catenin (right). Dashed lines indicate zero current. (b) Current–voltage relationship of *I*_Na_ in BrS iPSC‐CMs expressing β‐catenin shRNA (*n* = 9 cells from two batches of cell differentiation, red color) or control shRNA (*n* = 8 cells from two batches of cell differentiation, black color). *p < 0.05 versus control shRNA by two‐tailed *t*‐test. Error bars indicate standard errors of the mean for clarity. Right panel shows individual data points at −10 mV. Error bars indicate standard deviations. (C) *I*_Na_ recorded from BrS iPSC‐CMs with a bath solution that contained 100 mM Na^+^ (*E*_Na_ = +76 mV) without Cd^2+^ (instead, nifedipine was included to inhibit L‐type Ca^2+^ current). (a) Representative *I*_Na_ recorded from BrS iPSC‐CMs expressing control shRNA (left) or β‐catenin shRNA (right). Dashed lines indicate zero current. (b) Current–voltage relationship of *I*_Na_ in BrS iPSC‐CMs expressing β‐catenin shRNA (*n* = 14 cells from two batches of cell differentiation, red color) or control‐shRNA (*n* = 9 cells from two batches of cell differentiation, black color). Blue dots indicate values in healthy iPSC‐CMs (copied from the control group in Figure 1Eb) for comparison. *p < 0.01 by two‐tailed *t*‐test. Error bars indicate standard errors of the mean for clarity. Right panel shows individual data points at −20 mV. Error bars indicate standard deviations.

Consistent with western blot data, immunocytostaining also demonstrated a greater level of Na_v_1.5 in α‐SA‐positive cardiomyocytes in BrS iPSC‐CMs after β‐catenin knockdown (Figure 5A, middle panel). Co‐staining with N‐cadherin, a plasma membrane protein, demonstrated increased Na_v_1.5 on the plasma membrane of BrS iPSC‐CMs after β‐catenin knockdown (Figure 5A, bottom panel). The α‐SA protein is located at the Z‐discs of myofibrils in cardiomyocytes and the α‐SA staining indicated organized sarcomeres in BrS iPSC‐CMs of the control shRNA group suggesting a high level of maturation in these cells. Consistent with western blot data showing no changes in total α‐SA protein after β‐catenin knockdown, the organized α‐SA expression was present in BrS iPSC‐CMs of both control shRNA and β‐catenin shRNA groups (Figure 5A, middle panel). These observations provide further evidence that the maturation of the cells was not significantly affected by β‐catenin knockdown.

*I*_Na_ was recorded in BrS iPSC‐CMs with the whole‐cell patch‐clamp technique using a bath solution containing 100 mM Na^+^ (*E*_Na_ = +76 mV). *I*_Na_ was initially recorded in the presence of 0.1 mM Cd^2+^ (to block L‐type Ca^2+^ current) and *I*_Na_ was increased (*p* < 0.01) by 13‐fold in the cells after β‐catenin knockdown (−56.2 ± 33.7 pA/pF at −10 mV *n* = 9 cells vs. control‐shRNA −4.3 ± 4.6 pA/pF *n* = 8 cells, Figure 5B). To eliminate the *I*_Na_‐blocking effects of Cd^2+^, *I*_Na_ recording was repeated in the absence of Cd^2+^ (nifedipine was included to block L‐type Ca^2+^ current), and *I*_Na_ was increased (*p* < 0.01) by 4.9‐fold in the cells after β‐catenin knockdown (−107.4 ± 112.5 pA/pF at −20 mV *n* = 14 cells vs. control‐shRNA −21.8 ± 20.9 pA/pF *n* = 9 cells, Figure 5C). The smaller *I*_Na_ when Cd^2+^‐containing solution was used (Figure 5Bb vs. 5Cb) suggests that *I*_Na_ was partly inhibited by Cd^2+^, consistent with previous reports showing Cd^2+^ inhibition of cardiac *I*_Na_ with an IC_50_ of 0.18 mM (DiFrancesco et al., 1985). The reversal potential (*E*_rev_) of *I*_Na_ in control‐shRNA group (+70 mV) was close to *E*_Na_ (+76 mV) when Cd^2+^ was omitted (Figure 5Cb). However, the *E*_rev_ in control‐shRNA group was shifted to +30 mV when Cd^2+^ was present (Figure 5Bb) suggesting possible contamination of other currents in the whole‐cell recording when *I*_Na_ was inhibited by Cd^2+^.

Compared with *I*_Na_ recorded in healthy iPSC‐CMs under the same ionic conditions (−138.3 pA/pF at −20 mV, shown in both Figures 5Cb and 1Eb), there is an 84% reduction of *I*_Na_ in BrS iPSC‐CMs (−21.8 pA/pF at −20 pA/pF, control‐shRNA group in Figures 5Cb), which is consistent with a lower level of Na_v_1.5 in BrS iPSC‐CMs (Figure 3a). β‐catenin knockdown in BrS iPSC‐CMs increased *I*_Na_ (−107.4 pA/pF at −20 mV) to 78% of that in healthy iPSC‐CMs. This is consistent with observations in immunocytostaining that Na_v_1.5 is increased in the plasma membrane of cardiomyocytes in BrS iPSC‐CMs after β‐catenin knockdown.

### 3.6 β‐catenin knockdown increases Na_v_1.5 in a second line of Brugada syndrome iPSC‐CMs

**FIGURE 6:**
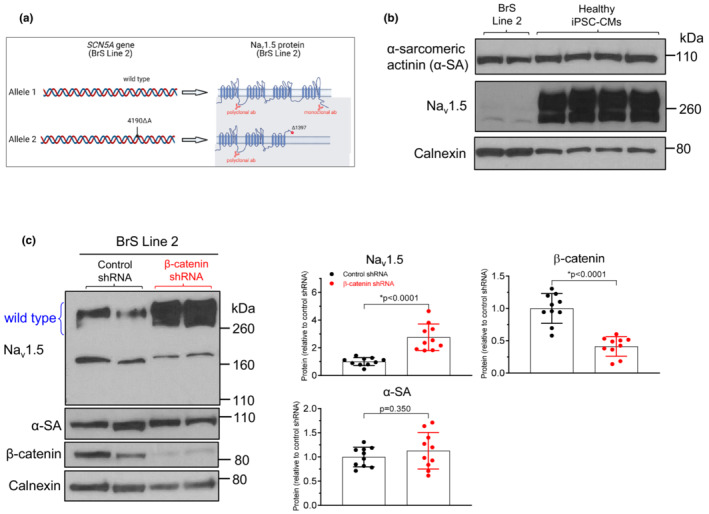
β‐catenin knockdown increases Na_v_1.5 protein in a second line of Brugada syndrome iPSC‐CMs. (a) Diagram of the genotype of a different line of Brugada syndrome iPSC‐CMs (BrS Line 2). This BrS cell line harbors heterozygous mutation. The mutant allele contains 1‐base pair deletion causing a frame shift mutation, 4190ΔA, in *SCN5A* gene which is anticipated to generate a truncated non‐functional Na_v_1.5. The monoclonal Na_v_1.5 antibody binds to the C‐terminus and will only detect the wild‐type Na_v_1.5. (b) Western blot showing similar level of α‐sarcomeric actinin (α‐SA, a marker of cardiomyocyte maturation), but reduced level of the wild‐type Na_v_1.5 (polyclonal antibody) in BrS Line 2 iPSC‐CMs as compared to healthy iPSC‐CMs. (c) Western blot showing upregulation of the full‐length, wild‐type Na_v_1.5 (the bands above 260 kDa) (using a monoclonal antibody) and unchanged α‐sarcomeric actinin (α‐SA) after shRNA‐mediated β‐catenin knockdown in BrS Line 2 iPSC‐CMs. Right panel: quantification of western blot data. *n* = 10 samples from two independent differentiations. Data were analyzed by two‐tailed *t*‐test.

To investigate if the upregulation of Na_v_1.5 by β‐catenin knockdown is reproduced in cardiomyocytes of other BrS patients, we tested an additional BrS iPSC‐CM line (BrS Line 2) that contains a mutation in one of the two *SCN5A* alleles (Figure 6a) as described by Liang et al. (Liang et al., 2016). The mutated allele contains a 1‐base pair deletion causing a frame shift mutation (4190ΔA) in *SCN5A* gene, which is anticipated to generate a truncated Na_v_1.5 protein starting at amino acid 1397 (located in the linker region between segments 5 and 6 of domain III, Figure 6a). Previous studies have shown that similar truncation mutations in this linker region (e.g., at amino acid 1393 or 1638) cause complete (100%) loss of channel function (Meregalli et al., 2009). Because the mutant, truncated Na_v_1.5 protein lacks the C‐terminal and intracellular loop between domains III and IV (Figure 6a), which are required for Na_v_1.5 expression on plasma membrane (Rook et al., 2012; Ziane et al., 2010), it is unlikely that the truncated channels will interact with wild‐type channels on the sarcolemma.

Our western blot studies showed that, similar to observations in BrS Line 1 (Figure 3a), BrS Line 2 iPSC‐CMs also have a lower level of full‐length, wild‐type Na_v_1.5 as compared to healthy control iPSC‐CMs (Figure 6b). However, BrS Line 2 iPSC‐CMs and healthy iPSC‐CMs had similar level of α‐sarcomeric actinin (α‐SA, Figure 6b), suggesting that they have a similar level of maturation. The lower level of Na_v_1.5 in BrS Line 2 iPSC‐CMs is consistent with previous RNA sequencing study by Liang et al. (Liang et al., 2016) that demonstrated a lower level of *SCN5A* transcript in these cells as compared to healthy control iPSC‐CMs. This suggests that reduced S*CN5A*/Na_v_1.5 level contributes to the pathogenesis in this BrS patient.

In BrS Line 2 iPSC‐CMs, the full‐length Na_v_1.5, encoded by the wild‐type *SCN5A* allele (Figure 6a), was increased by 2.7‐fold after β‐catenin knockdown (2.76 ± 0.96 *n* = 10, vs. control shRNA 1.00 ± 0.29 *n* = 10, *p* < 0.0001, Figure 6c). Consistent with observations in the first line of BrS iPSC‐CMs (BrS Line 1, Figure 4C), α‐SA was not affected by β‐catenin knockdown in BrS Line 2 iPSC‐CMs (*p* = 0.35, Figure 6C), suggesting that the increased Na_v_1.5 was not secondary to alterations in cell maturity.

## 4 DISCUSSION

The present study demonstrated that pharmacological activation of Wnt/β‐catenin signaling leads to downregulation of Na_v_1.5 in both healthy and BrS human iPSC‐CMs. In addition, the downregulation of Na_v_1.5 is observed in both male and female iPSC‐CMs suggesting that this is a conserved mechanism, consistent with the fact that the Wnt/β‐catenin signaling is a fundamental signal transduction pathway in the animal kingdom (Cadigan & Nusse, 1997). The downregulation of *SCN5A* mRNA after Wnt/β‐catenin signaling activation suggests that reduced *SCN5A* gene expression is the primary (if not the only) mechanism underlying Na_v_1.5 reductions. This aligns with the well‐established role of the Wnt/β‐catenin signaling as a regulator of gene transcriptions (Cadigan & Nusse, 1997).

In both male and female iPSC‐CMs, western blot analyses showed a higher band (above 260 kDa) and a lower band (near or slightly lower than 260 kDa) when a polyclonal anti‐Na_v_1.5 antibody was used. This may reflect the different levels of Na_v_1.5 glycosylation as suggested by previous studies that the higher band corresponds to a fully‐glycosylated, mature form, while the lower band corresponds to a partially‐glycosylated, immature form (Mercier et al., 2015). The higher‐to‐lower band ratio was reduced by CHIR in both male and female iPSC‐CMs, which suggests reduced Na_v_1.5 glycosylation and may explain the alterations in *I*_Na_ kinetics as observed in male iPSC‐CMs. The difference in the relative abundance of the higher and lower Na_v_1.5 bands in untreated cells may reflect the different channel glycosylation levels due to possible variations in cell maturation between male and female iPSC‐CMs or among the different batches of cell differentiations in each iPSC‐CM line. The greater reductions in total Na_v_1.5 in male iPSC‐CMs suggest that they are more sensitive to CHIR treatment than female iPSC‐CMs. Previous studies have shown a half‐life of 32–36 h for Na_v_1.5 in adult dog cardiomyocytes (Maltsev et al., 2008). The apparent unchanged lower Na_v_1.5 band in female iPSC‐CMs at 48 h after CHIR treatment may result from the combination of accelerated channel de‐glycosylation and accelerated channel degradation. An alternative explanation of the lower Na_v_1.5 band is that it is a partly degraded Na_v_1.5 protein missing the C‐terminus, because this band was not detected when a monoclonal anti‐Na_v_1.5 antibody targeting the C‐terminus was used. However, the validity of this alternative explanation would require the verification of the epitope specific binding of the monoclonal antibody.

This study further demonstrated that inhibition of the Wnt/β‐catenin signaling, using two different strategies, upregulated Na_v_1.5 in BrS iPSC‐CMs suggesting that this is a potential therapeutic strategy. The increased *I*_Na_ density, as measured in single cells, is consistent with immunocytostaining results showing upregulation of Na_v_1.5 proteins at the plasma membrane in individual cardiomyocytes after β‐catenin knockdown. This also suggests that the increased total Na_v_1.5 protein in BrS iPSC‐CMs after β‐catenin knockdown, as demonstrated by western blot, reflects increased Na_v_1.5 expression in the individual cardiomyocytes, instead of increased number of Na_v_1.5‐expressing cells due to altered cell proliferation or survival. Despite the clear and consistent upregulation of Na_v_1.5 protein, the *SCN5A* mRNA was, surprisingly, either not increased or increased to a smaller degree after Wnt signaling inhibition. This suggests that, in addition to the known effect of Wnt signaling on *Scn5a* transcription (Lu et al., 2020; Wang et al., 2016), Wnt/β‐catenin signaling may also regulate Na_v_1.5 at the posttranscriptional or posttranslational level. In addition to its well‐known role as the intracellular mediator of the Wnt/β‐catenin pathway, β‐catenin also plays a role in cell–cell junction (Valenta et al., 2012). Most of the β‐catenin protein in a cell is located in the cytoplasmic side of the plasma membrane forming a complex with cadherin and other binding partners (Valenta et al., 2012). Our immunocytostaining of BrS iPSC‐CMs showed that most β‐catenin is co‐localized with N‐cadherin (Figure 5A, top panel), a plasma membrane protein expressed in cardiomyocytes (Zuppinger et al., 2000). This plasma membrane pool of β‐catenin was also reduced by β‐catenin shRNA (Figure 5A). Because direct interactions between Na_v_1.5 and N‐cadherin have been demonstrated in cardiomyocytes (Leo‐Macias et al., 2016), it is possible that β‐catenin knockdown affects the binding of Na_v_1.5 to N‐cadherin and other binding partners on the plasma membrane (Shy et al., 2013), leading to increased Na_v_1.5 stability and a slower degradation rate. In addition, calnexin, a chaperon protein in the endoplasmic reticulum that is important for protein folding and N‐glycosylation (Kozlov & Gehring, 2020), appeared to be altered after Wnt signaling modulations. Future studies to quantify the changes in calnexin and their potential role in Na_v_1.5 synthesis and tracking to the plasma membrane will show if this is one of the mechanisms for the regulation of Na_v_1.5 by Wnt/β‐catenin signaling.

Wnt/β‐catenin signaling is known to regulate embryonic cardiogenesis and iPSC differentiation (Burridge et al., 2014; Gessert & Kuhl, 2010; Lian et al., 2012). It is required for the induction of cardiac mesoderm (Lindsley et al., 2006; Yamaguchi et al., 1999), but it inhibits the differentiation of cardiac mesoderm cells into cardiomyocytes (Naito et al., 2006; Ueno et al., 2007) and the maturation of early‐stage cardiomyocytes (at Day 12 of iPSC differentiation) (Buikema et al., 2020). The present study investigated late‐stage iPSC‐CMs (at Day 30–45 of differentiation) that have matured in culture, as indicated by the organized sarcomere protein expression in the cells. The CHIR‐induced reductions in *SCN5A* mRNA are highly correlated with the levels of Wnt signaling activation as estimated by *AXIN2* mRNA increases. In addition, we and others have previously demonstrated downregulation of *Scn5a*/Na_v_1.5 in adult mouse and rat ventricular myocardium after activation of Wnt/β‐catenin signaling (Huo et al., 2019; Lu et al., 2020; Wang et al., 2021). A more recent study (after the preprint posting of the present study http://dx.doi.org/10.2139/ssrn.3815857) also showed downregulation of Na_v_1.5 in healthy adult human heart slices after treatment with a GSK‐3β inhibitor (SB216763) (Li et al., 2022). These observations suggest that the CHIR‐induced reductions in *SCN5A/Nav1.5* in iPSC‐CMs are unlikely secondary to alterations in cardiomyocyte maturation. On the other hand, the upregulation of Na_v_1.5 in BrS iPSC‐CMs after inhibition of Wnt signaling is not accompanied by changes in maturation marker levels or sarcomere organization, suggesting that this effect is not mediated by altered cell maturation.

One limitation of the present study is that the iPSC‐CMs generated using standard protocols contain different types of cardiomyocytes (i.e., atrial, ventricular and nodal myocytes) (Hamel et al., 2017), which may explain the variation in Na_v_1.5 expression and *I*_Na_ density among the individual cells in this study. However, our previous study (Liang et al., 2015) using pure culture of neonatal rat ventricular myocytes (NRVMs) and other studies using mouse atrial cell line (Wang et al., 2016; Zhao et al., 2019), are consistent with findings in iPSC‐CMs of this study, suggesting that the key findings can be applied to both atrial and ventricular myocytes. The second limitation of iPSC‐CMs is their immature phenotype (Hamel et al., 2017; Liang et al., 2019). Although the present study used relatively mature iPSC‐CMs (at 30–45 days of differentiation), future studies are needed to further investigate the effects of Wnt signaling inhibition on adult BrS cardiomyocytes before advocating it as a therapeutic strategy in the patients.

In summary, this study demonstrated downregulation of Na_v_1.5 by Wnt/β‐catenin signaling in both male and female iPSC‐CMs with reduced *SCN5A* mRNA as one of the underlying mechanisms. More importantly, this study showed that blocking Wnt/β‐catenin signaling is a valid strategy to restore the expression of Na_v_1.5 and voltage‐gated Na^+^ current in iPSC‐CMs of BrS patients.

## AUTHOR CONTRIBUTIONS

Conception or design of the work: A.L., D.R.D., and W.L.; Acquisition, analysis or interpretation of data for the work: A.L., R.G., C.C., Y.X., J.W., and W.L.; Drafting the work or revising it critically for important intellectual content: A.L., R.G., C.C., Y.X., J.W., D.R.D., and W.L. All authors have approved the final version of the manuscript, agreed to be accountable for all aspects of the work in ensuring that questions related to the accuracy or integrity of any part of the work are appropriately investigated and resolved, and all persons designated as authors qualify for authorship, and all those who qualify for authorship are listed.

## FUNDING INFORMATION

This work was supported by Canadian Institutes of Health Research (PJT‐148918, to W.L.), McDonald Scholarship and New Investigator Award from Heart and Stroke Foundation of Canada (to W.L.), University of Ottawa Faculty of Medicine Translational Research Grant (to D.R.D. and W.L.), Endowed Fellowship (to A.L.) and Endowed Scholarship (to Y.X. and J.W.) from the University of Ottawa Heart Institute.

## CONFLICT OF INTEREST STATEMENT

The authors have no competing interest.
